# Limited alignment of publicly competitive disease funding with disease burden in Japan

**DOI:** 10.1371/journal.pone.0228542

**Published:** 2020-02-10

**Authors:** Shuhei Nomura, Daisuke Yoneoka, Shiori Tanaka, Ryoko Makuuchi, Haruka Sakamoto, Aya Ishizuka, Haruyo Nakamura, Anna Kubota, Kenji Shibuya

**Affiliations:** 1 Department of Global Health Policy, Graduate School of Medicine, The University of Tokyo, Tokyo, Japan; 2 Department of Health Policy and Management, School of Medicine, Keio University, Tokyo, Japan; 3 Epidemiology and Prevention Group, Center for Public Health Sciences, National Cancer Center, Tokyo, Japan; 4 Graduate School of Public Health, St. Luke's International University, Tokyo, Japan; 5 Faculty of Medicine, Charles University, Hradec Kralove, Czech Republic; Public Health Foundation of India, INDIA

## Abstract

**Objective:**

The need to align investments in health research and development (R&D) with public health needs is one of the most important public health challenges in Japan. We examined the alignment of disease-specific publicly competitive R&D funding to the disease burden in the country.

**Methods:**

We analyzed publicly available data on competitive public funding for health in 2015 and 2016 and compared it to disability-adjusted life year (DALYs) in 2016, which were obtained from the Global Burden of Disease (GBD) 2017 study. Their alignment was assessed as a percentage distribution among 22 GBD disease groups. Funding was allocated to the 22 disease groups based on natural language processing, using textual information such as project title and abstract for each research project, while considering for the frequency of information.

**Results:**

Total publicly competitive funding in health R&D in 2015 and 2016 reached 344.1 billion JPY (about 3.0 billion USD) for 32,204 awarded projects. About 49.5% of the funding was classifiable for disease-specific projects. Five GDB disease groups were significantly and relatively well-funded compared to their contributions to Japan’s DALY, including neglected tropical diseases and malaria (funding vs DALY = 1.7% vs 0.0%, p<0.01) and neoplasms (28.5% vs 19.2%, p<0.001). In contrast, four GDB disease groups were significantly under-funded, including cardiovascular diseases (8.0% vs 14.8%, p<0.001) and musculoskeletal disorders (1.0% vs 11.9%, p<0.001). These percentages do not include unclassifiable funding.

**Conclusions:**

While caution is necessary as this study was not able to consider public in-house funding and the methodological uncertainties could not be ruled out, the analysis may provide a snapshot of the limited alignment between publicly competitive disease-specific funding and the disease burden in the country. The results call for greater management over the allocation of scarce resources on health R&D. DALYs will serve as a crucial, but not the only, consideration in aligning Japan's research priorities with the public health needs. In addition, the algorithms for natural language processing used in this study require continued efforts to improve accuracy.

## Introduction

The need to align investments in health research and development (R&D) with the public health needs is one of the most important public health challenges in Japan, as in other countries and the world [1–10]. According to the data from the Organisation for Economic Co-operation and Development (OECD), Japan's R&D expenditures (in all sectors, not just the health sector) are comparable to those of OECD countries. In 2017, Japan ranked 3rd at 155.1 billion USD after the United States (US) (483.7 billion USD) and China (442.7 billion USD) in R&D expenditure; and as a percentage of gross domestic spending (GDP), it ranks 5th at 3.2% after South Korea (4.6%), Israel (4.5%), Sweden (3.3%) and Chinese Taipei (3.3%) [11]. In Japan, public R&D funding is mostly based on competitive funding systems administrated separately by the Ministry of Education, Culture, Sports, Science and Technology (MEXT) or other ministries or government agencies. The amount of funds allocated to researchers, according to Science and Technology Basic Plan, is mainly based on scientific or technical evaluation [12]. However, the allocation largely follows the previous resource allocation and may not necessarily reflect disease burden priorities in the country [13].

With the rapid aging of the population, the shift of the disease pattern from acute towards chronic and age-related illnesses (multimorbidity), and rising health care costs, many countries and international agencies are using disease burden (namely, disability-adjusted life year: DALYs) as an important indicator to identify and understand key public health demands and prioritize health policy concerns for resource allocation, interventions, service provision, research, and advocacy [14, 15]. DALY is the most comprehensive measure of population health to date, which considers both the death and disability associated with a given disease [16]. However, there has not been sufficient discussion in Japan on R&D prioritization based on disease burden, except for our previous studies [17].

In order to efficiently carry out health R&D, it is essential to grasp the burden of disease in the nation, decide the priority of development, and respond strategically. This study aimed to examine the alignment of disease-specific public R&D funding to DALYs in Japan, using the data of public competitive research funding systems. Given some of the funding is clearly intended for a global health context, the alignment with global DALYs were also performed as a sub-analysis.

## Materials and methods

### Japan’s and global DALYs

We used the estimates of DALYs for Japan and the world for the year of 2016, published by the Global Burden of Disease Study (GBD) 2017 [18]. The period was determined by the availability of data of health R&D funding (section below). DALY combines in one measure the time lost due to individuals' premature death from each disease/injury and the time lived with disability, taking into account the degree of severity of disability associated with different states of poor health caused by each disease/injury [16]. Using published studies and available data worldwide, the GBD’s latest study (GBD2017) covered 195 countries and territories, and estimated DALYs and other health metrics for 359 diseases and injuries for each year from 1990 until 2017. The results of the GBD study have been widely used by researchers, policy-makers, and several other stakeholders to argue for more strategic resource allocation [19–26].

Details of the methods for estimating DALYs are provided in the capstone publication of the GBD 2017 study for DALYs [27]. Each of 359 diseases and injuries were arranged in a 4-level mutually exclusive and collectively exhaustive cause hierarchy set by the GBD; where most of the diseases were analyzed for causing both death and disability. In order to draw an easier policy interpretation, we employed the second level of the cause list, comprising of 22 disease groups under three major groupings enlisted in the first level: communicable, maternal and neonatal conditions, and nutritional deficiencies; non-communicable diseases; and injuries. The full GBD disease list, including corresponding codes from the 10^th^ revision of International Classification of Diseases (ICD-10) (i.e. GBD-ICD mapping tables), is detailed in appendices to the GBD 2017 summary publications [28, 29].

### Health R&D funding in Japan

In this study, we considered the databases of three major public competitive funding systems, administrated by MEXT (and its affiliated agency, the Japan Society for the Promotion of Science), the Ministry of Health and Labour Welfare of Japan (MHLW), and the Japan Agency for Medical Research and Development (AMED). AMED is a recently created cross-ministry agency in charge of all state health research projects. Researchers belonging to domestic research institutions are eligible to apply for funding from these agencies. The databases of these agencies are (1) Database of Grants-in-Aid for Scientific Research; (2) MHLW Grants System; and (3) AMEDfind, respectively [30–32]. They are free and open access databases. For the sake of simplicity, these databases are defined as ‘MEXT’, ‘MHLW’ and ‘AMED’ hereafter in this study. The study period was from January 2015 to December 2016, based on the availability of data. With MEXT in particular, we used only data indexed in the research fields (‘saimoku’ in Japanese) of medicine, dentistry, and pharmacy as the MEXT funding system covers all science fields, not just in the field of health.

Given the scope of this study, we did not consider any Japanese funding for global health R&D channeled through international research institutions and agencies (including official development assistance for health), which generally aim at the public health needs of low- and middle-income countries. In addition, research funding from international and foreign funders that were awarded through Japanese research institutions and researchers, was not considered.

The estimation of the health R&D allocation by 22 GBD disease groups was carried out in the following four steps.

#### Step 1: Text information extraction

For each research project implemented during 2015 and/or 2016, "year", "research funding", "research field", "project title", "abstract/keywords", "results", or similar items were obtained from each funding systems in a text format. Note that in the publicly competitive funding systems, research funding is awarded on a per-project basis. Even if multiple studies are generated from a single project, detailed information about individual studies (number and amount of expenses per study) is not available from the databases.

For MEXT and MHLW, we extracted the data ourselves using the download function. Since AMEDfind does not have a download function, we used AMED’s data extraction service to obtain data. Available text information in each database is listed in Table 1.

**Table 1:** Available text information in each database.

Funding system	Available information					
	Year	Total funding	Funding per year	Project title	Abstract/keywords	Results
MEXT	✓	✓	✓	✓	✓	✓
MHLW	✓	✓		✓	✓	✓
AMED	✓		✓	✓	✓	

MEXT: Ministry of Education, Culture, Sports, Science and Technology; MHLW: Ministry of Health and Labour Welfare; AMED: Japan Agency for Medical Research and Development.

#### Step 2: ICD 10 code mapping

From the collected text information of each research project, disease and injury names were extracted using natural language processing, and ICD 10 codes corresponding to them were assigned. Natural language processing is a technique that extracts useful knowledge from a vast amount of natural language text data.

Standard Disease-Code Master (SDCM)[33] was used for the list of disease and injury name to be extracted and the correspondence table of ICD 10. SDCM is the most widely-used, open-access diagnostic classification standard for clinical and research purposes in Japan. The current version of SDCM covers approximately 25,938 disease/injury names, where each name is assigned to a corresponding ICD 10 code. The SDCM was first developed in 2001 by the Medical Information System Development Center (MEDIS-DC) commissioned by MHLW, and has been updated periodically [34]. Because SDCM is a well-defined list, text preprocessing, such as elimination of stop words, text normalization (stemming and lemmatization), and standardization of spelling variations, was not required.

As a result, ICD 10 code(s) were assigned to each research project. Research projects that were assigned at least one ICD 10 code were defined as disease-specific research projects. If the disease/injury names listed in the SDCM were not present in the text information of a research project and no ICD 10 code was assigned to the project, it was considered unclassifiable, and therefore categorized as a non-disease-specific research project.

#### Step 3: GBD disease group mapping

Following the ICD code mapping, the 22 GBD disease groups were mapped to the ICD 10 codes assigned to the projects, based on the GBD-ICD mapping tables [28, 29]. The GBD-ICD mapping tables were originally developed by the GBD 2017 for diseases causing deaths and disability, separately [28, 29]. Because this study does not take into account the differences between death and disability due to the nature of estimating the funding by disease groups, we created a combined GBD-ICD mapping table for our research purposes, simply by merging these tables for death and disability.

More importantly, the ICD 10 codes include several unusable codes (not useful) for mortality coding, which is widely acknowledged as the so-called ‘garbage code’ (GC). There is a variable use of these GCs [35, 36]. For example, as part of the estimation process of DALYs in the GBD study, all deaths assigned to GC are proportionately re-distributed to all GBD cause groups through statistical models and algorithms for each age-sex-location-year group [28]. Meanwhile, for our purpose of estimating funding by GBD disease groups, we handled GC by re-assigning them to the most probable underlying GBD disease group, based on expert opinion and knowledge about the disease/injuries. The complete GBD-ICD mapping table developed for this study can be found in S1 Table. We did not consider the following ICD 10 codes in this study: R00–R99 (symptoms, signs and abnormal clinical and laboratory findings, not elsewhere classified); Z00–Z99 (factors influencing health status and contact with health services); U00–U99 (codes for special purposes); and Y10–Y34 (event of undetermined intent).

#### Step 4: Weighting and re-distributing into 22 GBD disease groups

As a result, GBD disease group(s) were assigned to each disease-specific research project. For the projects with several different disease groups assigned, we considered the top two frequent disease groups as its main disease group (most frequently assigned GBD disease group) and a sub disease group (second most frequently assigned GBD disease group). For example, if ‘neoplasms’ is the most frequent disease group in the text information of a project, followed by ‘cardiovascular diseases’, then the project's main disease group is ‘neoplasms’ and its secondary disease is categorized as ‘cardiovascular diseases’. If there is no difference in the assigned frequency of the top two disease groups of a research project, the frequency within “project title”, “abstract/keywords", and "result" were considered in this order to determine which GBD disease groups should be the main disease group or the sub disease group. If there was still no difference, we considered the disease group that appeared first in the text information as the main group.

We then weighted the main and sub disease groups for each database, and the funding of each disease-specific research project was re-distributed to the 22 GDB disease groups accordingly. The weights parameters *α*_*jk*_ for main (*j* = 1) and sub (*j* = 2) GBD disease groups in *k* database (i.e. MEXT (*k* = 1), MHLW (*k* = 2), AMED (*k* = 3), and Total (*k* = 4: MEXT, MHLW, and AMED combined)) were estimated based on the following regression model:

$$y_{ik}=\sum_{j=1}^{2}\alpha_{jk}x_{ijk}+\varepsilon_{ik},\;\text{s}.\text{t}.\sum_{j=1}^{2}\alpha_{jk}=1\;\text{and}\;\alpha_{jk}\geq 0,$$ (1)

where *y*_*ik*_ is the standardized amount of funding *Y*_*ik*_ of *i* research project in *k* database, *x*_*ijk*_ = {1,2,…, 23} (i.e. 22 GDB disease groups plus “unclassifiable”) indicates the main or sub GDB disease groups of *i* research project in *k* database, and $\varepsilon_{ik}$ is an error term of *i* research project in *k* database. The standardization was performed using the following formula:

$$y_{ik}=\left(Y_{ik}-\mu_{K}\right)/\sigma_{k},$$

where *μ*_*K*_ and σ_*k*_ were mean and standard deviation of *Y*_*ik*_, respectively. The *α*_*jk*_ were estimated using a least squared method with the constraints [37, 38]. Based on the estimates of *α*_*jk*_, which were denoted by ${\hat{\alpha }}_{jk}$, the (unstandardized) amount of funding for main or sub GDB disease groups ${\hat{F}}_{ijk}$ were calculated (re-distributed) using the following formula:

$${\hat{F}}_{ijk}={\hat{\alpha }}_{jk}Y_{ik}$$

The redistributed funding of each research project were then summed up in the 22 groups. Note that if a research project had only one GDB disease group, ${\hat{\alpha }}_{1}=1$ and ${\hat{\alpha }}_{2}=0$ were used as weights.

#### Alignment of publicly competitive disease-specific R&D funding to disease burden

Lastly, the distribution of disease-specific R&D for 2015 and 2016 by 22 GBD disease groups was compared with that of DALYs in 2016. To test the statistical association between the funding and DALYs, two-proportion z test (two-sided) with Bonferroni correction for multiple testing was performed. All analyses used R version 3.6.0 software (R Core Team), and p < 0.05 was considered to denote statistical significance. The USD/JPY exchange rate was the average of the target years (i.e. 114.92 JPY = 1 USD) as reported by the OECD [39]. Because Japan has a low inflation rate (less than 1%) and does not take inflation into account in the budgeting of research funding, we used a constant value for this study.

### Ethical approval

Ethical approval was not sought for the present study because human subjects were not included.

## Results

**Fig 1:**
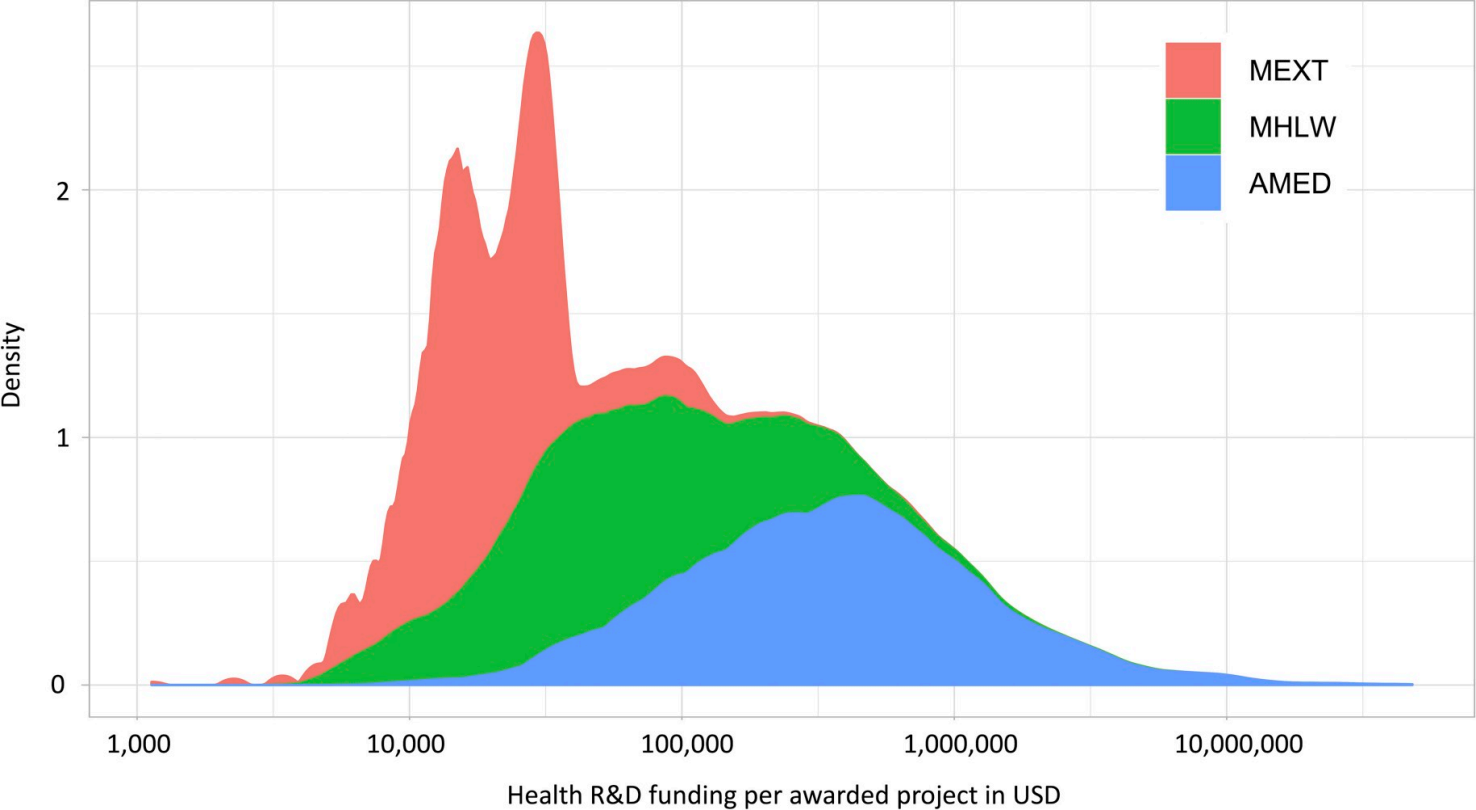
Stacked (Kernel) density plot of health R&D funding per awarded project of each funding system (2015–2016). X-axis is log10 scaled. MEXT: Ministry of Education, Culture, Sports, Science and Technology; MHLW: Ministry of Health and Labour Welfare; AMED: Japan Agency for Medical Research and Development.

From 2015 to 2016, we identified 32,204 awarded research projects; whose allocated funding amount totaled 344.1 billion JPY (approx. 3.0 billion USD). For those research projects spanning over two years, they were counted as one awarded project. AMED funded research accounted for 71.9% of the total funding, followed by MEXT for 24.8%. AMED had the largest research funding per project at about 0.8 (standard deviation 2.1) million USD on average, followed by MHLW at 0.1 (2.1) million USD. The detailed characteristics by funding systems are shown in Table 2. Also, Fig 1 shows a kernel density plot (Epanechnikov kernel) of health R&D funding for 2015 and 2016, indicating that the allocation of funding per awarded project is not particularly skewed to high or low, while AMED is spending a relatively larger sum per research project.

**Table 2:** Number of the awarded research project for 2015–2016 and amount of health R&D funding by the publicly competitive funding system.

Funding system	Number of awarded project	Funding in JPY (million)	Funding in USD* (million)	% of total funding	Mean project funding (SD) in USD* (thousand)
MEXT	28,806	85,355.1	742.7	24.8	25.8 (35.1)
MHLW	849	11,255.5	97.9	3.3	115.4 (214.0)
AMED	2,549	247,504.8	2,153.7	71.9	844.9 (2,136.4)
Total	32,204	344,115.4	2,994.4	100.0	93.0 (642.1)

* 114.92 JPY = 1 USD. SD: standard deviation. MEXT: Ministry of Education, Culture, Sports, Science and Technology; MHLW: Ministry of Health and Labour Welfare; AMED: Japan Agency for Medical Research and Development.

Out of 32,204 awarded projects, 11.4% (n = 3,674) had several different GBD disease groups assigned, while 38.1% (n = 12,257) were not assigned to any GBD disease groups (i.e. non-disease-specific research project). The estimated allocation weights in Eq 1 are ${\hat{\alpha }}_{1}$ = 0.559 and ${\hat{\alpha }}_{2}$ = 0.442 for the total funding of the three systems (see S2 Table for more details). Based on the weights, Table 3 shows the estimated total health R&D funding (MEXT, MHLW, and AMED combined) for 2015–2016 by the 22 GBD disease groups. About 49.5% of health R&D was classifiable to disease-specific projects. In other words, unclassifiable funding of the remaining 50.5% may be allocated to non-disease-specific projects, such as basic science research. Out of the 22 GBD disease groups, neoplasms received the largest amount of funding at 422.4 million USD (14.1%). Neurological disorders received 179.6 million USD (6.0%), followed by other non-communicable diseases (149.7 million USD (5.0%)), unintentional injuries (126.4 million USD (4.2%)), cardiovascular diseases (117.8 million USD (3.9%)), and digestive diseases (114.0 million USD (3.8%)). Funding system-specific results, which were similar to the combined results (except for neoplasms in MHLW), can be found in S3–S5 Tables.

**Table 3:** The estimated total health R&D funding (2015–2016) by the 22 GBD disease categories.

GBD disease groups at level 1	GBD disease groups at level 2	Funding in JPY (million)	Funding in USD* (million)	% of total funding
Communicable, maternal and neonatal conditions and nutritional deficiencies	1. HIV/AIDS and sexually transmitted infections	335.3	2.9	0.1
	2. Respiratory infections and tuberculosis	6,725.8	58.5	2.0
	3. Enteric infections	1,054.3	9.2	0.3
	4. Neglected tropical diseases and malaria	2,933.0	25.5	0.9
	5. Other infectious diseases	5,886.6	51.2	1.7
	6. Maternal and neonatal disorders	876.5	7.6	0.3
	7. Nutritional deficiencies	64.5	0.6	0.0
Non-communicable diseases	8. Neoplasms	48,539.6	422.4	14.1
	9. Cardiovascular diseases	13,532.6	117.8	3.9
	10. Chronic respiratory diseases	2,481.6	21.6	0.7
	11. Digestive diseases	13,103.3	114.0	3.8
	12. Neurological disorders	20,636.2	179.6	6.0
	13. Mental disorders	8,622.7	75.0	2.5
	14. Substance use disorders	284.4	2.5	0.1
	15. Diabetes and kidney diseases	3,484.3	30.3	1.0
	16. Skin and subcutaneous diseases	3,318.6	28.9	1.0
	17. Sense organ diseases	4,769.5	41.5	1.4
	18. Musculoskeletal disorders	1,619.4	14.1	0.5
	19. Other non-communicable diseases	17,208.6	149.7	5.0
Injuries	20. Transport injuries	-	-	0.0
	21. Unintentional injuries **	14,520.9	126.4	4.2
	22. Self-harm and interpersonal violence	190.8	1.7	0.1
	23. Unclassifiable	173,927.0	1,513.5	50.5

* 114.92 JPY = 1 USD;

** Unintentional injuries do not include transport injuries. Other infectious diseases include meningitis, encephalitis, diphtheria, whooping cough, tetanus, measles, varicella and herpes zoster, acute hepatitis, and other unspecified infectious diseases; other non-infectious diseases include congenital birth defects, urinary diseases and male infertility, gynecological diseases, hemoglobinopathies and hemolytic anemias, endocrine, metabolic, blood, and immune disorders, oral disorders, and sudden infant death syndrome.

### Mapping of disease-specific R&D funding and DALYs

**Fig 2:**
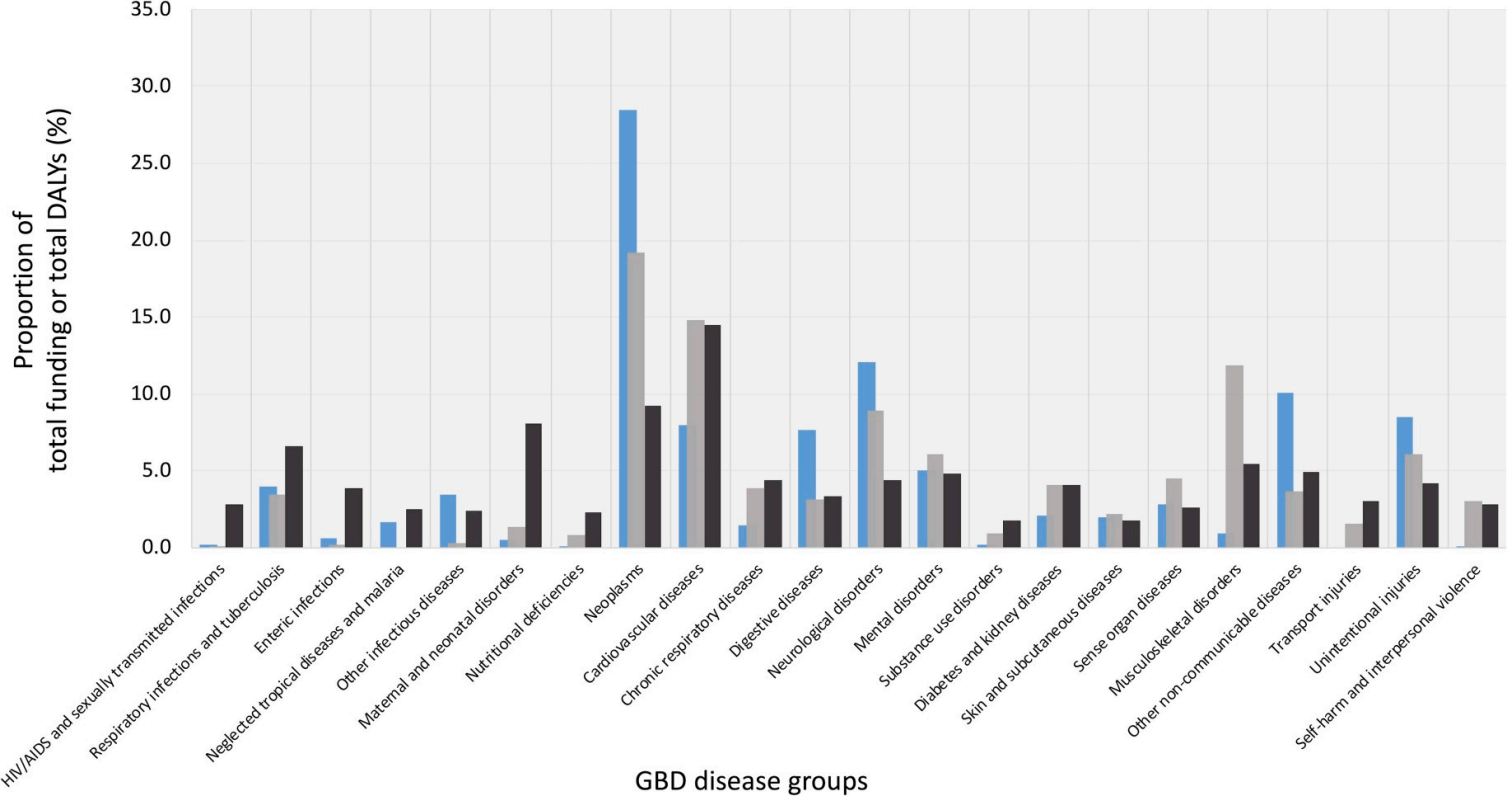
Balance of total health R&D funding and Japan’s and global DALYs. Gray: Japan’s DALYs; Black: Global DALYs; Blue: disease-specific R&D funding for 2015 and 2016. Proportion does not include ‘unclassifiable’. Unintentional injuries do not include transport injuries.

Fig 2 illustrates the balance between the health R&D funding (2015–2016) and Japan’s and global DALYs in 2016 by the 22 GDB disease groups respectively. The following five GDB disease groups were significantly relatively well-funded compared to their contributions to Japan’s DALY: neglected tropical diseases and malaria (funding vs DALY = 1.7% vs 0.0%, p<0.01), other infectious diseases (3.5% vs 0.3%, p<0.001), neoplasms (28.5% vs 19.2%, p<0.001), digestive diseases (7.7% vs 3.1%, p<0.001), and other non-communicable diseases (10.1% vs 3.7%, p<0.001). These percentages do not consider unclassifiable funding. In contrast, the following four GDB disease groups were significantly underfunded in comparison with their contributions to Japan’s DALY: cardiovascular diseases (8.0% vs 14.8%, p<0.001), musculoskeletal disorders (1.0% vs 11.9%, p<0.001), transport injuries (0.0% vs 1.6%, p<0.05), and self-harm and interpersonal violence (0.1% vs 3.0%, p<0.001). A comparison with the global DALYs showed that Japan allocated a relatively large amount of R&D funding to neglected tropical diseases and malaria and other infectious diseases, which had little DALYs in Japan. Exact values are presented in S6 Table. Funding system-specific results, which were similar to the combined results (except for neoplasms in MHLW), and their exact values are presented in S1–S3 Figs and S7–S9 Tables, respectively.

## Discussion

Our analysis of Japan’s burden of disease data revealed that there is a limited alignment between current levels of disease-specific R&D funding from the public competitive funding systems and disease burden in Japan. The funding level for diseases like neglected tropical diseases and malaria, other infectious diseases, neoplasms, digestive diseases, and other non-communicable diseases was relatively high compared to their contributions to DALYs. In contrast, the funding level for cardiovascular diseases, musculoskeletal disorders, transport injuries, and self-harm and interpersonal violence was relatively low in comparison with their contributions to DALYs. Similar limited alignment of health R&D funding and disease burden has also been reported in other countries, such as the United Kingdom (UK) and India, regardless of the source of the funding and whether it is in the domestic or global context, is not necessarily consistent [5–7].

In this study, it is not clear why particular disease groups remain under- or well-funded relative to disease burden. In Japan, there are no clear criteria or guidelines for prioritizing and determining the allocation of publicly competitive R&D funding. The reasons why neoplasms were relatively well-funded may be related to the fact that Japan is actively developing anticancer drugs, molecularly targeted drugs, and immunotherapy, and that there is a growing momentum for elucidation of the etiology of neoplasms [40]. On the other hand, the reason why cardiovascular diseases was relatively under-funded may reflect a relatively low level of political interest and research activity for the diseases, despite the fact that cardiovascular diseases is one of the top two major cause of DALYs along with neoplasms in the country.

In Japan, after the Cancer Control Act was enforced in 2007, the Act on Promotion of Cancer Registries was enacted in 2013. According to the Act, a new system ‘National Cancer Registry’ was launched in 2016 with the aim to collect, analyze and manage data on all people diagnosed with cancer in Japan [41]. However, as for cardiovascular diseases, although surgical cases have been registered in the National Clinical Databases (NCD) since 2011 with major support from the Japan Surgical Society (JSS) [42], there is no disease-based registry system, leaving the exact number of patients with ischemic heart disease, stroke, aortic aneurysm, etc. in the country in the dark. Although symptomatic drugs, such as vasodilators and antihypertensive agents have been developed, there is a strong need to develop fundamental therapeutic and preventive agents.

From a global DALYs’ perspective, the allocation of publicly competitive health R&D funding for neglected tropical diseases (including malaria), for which the domestic DALYs is about zero, is reasonable. A similar trend is also observed in the UK and India. While the UK has a domestic DALYs distribution similar to that of Japan, Head et al. (2016) identified that research investments awarded to UK institutions from public and international funders demonstrated relative research strengthens in some neglected tropical diseases in light of the global DALYs’ context [6]. This indicates that neglected tropical diseases are an important area of UK’s global health policy; the UK is known to be the second largest investors in global R&D for neglected tropical diseases in the world [43]. The same reasoning may apply to Japan. In recent years, Japanese government has been increasingly placing more emphasis on neglected tropical diseases [43]. A notable example is the establishment of Global Health Innovative Technology Fund (GHIT Fund) led by Japan in 2013, a public-private partnership to support the development of medicines, vaccines and diagnostic technologies for neglected tropical diseases. Similarly, a recent study in India also found that neglected tropical diseases and three major infectious diseases (HIV/AIDS, tuberculosis, and malaria) accounted for higher R&D funding as compared with their contribution to the disease burden [7]. The authors of the said study explained that these diseases are prioritized in the national vertical disease control programs.

Examples of important criteria on resource allocation might include research quality, scientific innovation and opportunity, portfolio diversification, infrastructure building, transmissibility or population risk, collateral benefits to disease control, and public interest [44, 45]. Given these potential contributors to decision-making about funding allocation, perfect alignment with DALYs or any other measure of disease burden would not be expected. However, faced with competing priorities for scarce resources on health research, DALYs, a comprehensive health measure that reflects the aging of the population and public health needs, should be considered as an important benchmark in assuming research priorities for funding decisions [2, 3, 5, 46–50]. For instance, after a long discussion on what indicators to consider in the US [44, 51, 52], which is the largest investor in health R&D in the world [11]; the National Institutes of Health (NIH) noted in its recent strategic plan that DALYs would serve as a crucial consideration in aligning NIH’s research priorities with public health needs [53].

### Limitation

Our analyses were subject to several limitations. First, while DALY is an important population health measure, widely used as an important benchmark for health policy benchmark, its limitations are well-discussed in previous literatures [54]. In particular, DALYs cannot clearly indicate how much investment is needed for R&D, since such indications would need to weigh the science and technological development as well as funding opportunities, not simply the disease/injury or number of patients.

Second, non-disease-specific research accounted for 50.5% of publicly competitive R&D funding where it was difficult to credit specific disease for much of these research projects, contributing uncertainty to the analysis and reducing correlations between funding levels and disease burden [3]. Non-disease-specific research may include, for example, basic science research, social medicine research, human resource development, as well as development of infrastructure and systems (e.g. translation of basic research to practical application; development of environment for promoting practical use of robots, artificial intelligence (AI), and information and communications technology (ICT); and enhancement of auditing functions to ensure compliance with ethics, laws and guidelines) [55].

Third, the disease burden used in this study is the current one, and future disease risk was not considered. For example, the results of this study showed that funding for other infectious diseases is relatively high in view of its contribution to disease burden. However, considering that immigration and inbound tourism will increase in Japan in the future with the recent revision of the Immigration Control and Refugee Recognition Act, the risk of future infectious diseases may increase in Japan [56]. It would, then, be reasonable to invest more in infectious diseases as a precaution. Antibiotic resistance has spread to a number of infectious diseases and has also become one of the greatest threats to public health [57].

Fourth, this study did not consider research funding from international funders nor funding for global health R&D channeled through international research institutions and agencies because of the primary purpose of comparing funding of grants with domestic DALYs. However, as mentioned above, Japan allocated a relatively large amount of funding to neglected tropical diseases compared to their contributions to domestic DALYs. As such, public research funding and R&D strategies are not necessarily limited to domestic public health issues. Promotion of overseas expansion of Japanese medical care (such as the Asia Health and Human Well-Being Initiative) and other international cooperation and collaboration are also important subjects of research and development [58]. Unfortunately, there was no data to determine whether or not research project was globally oriented or not.

Fifth, in this study, the data of AMED, which provides the largest amount of publicly competitive health R&D funding, were available only in 2015 and 2016 (as it was recently created in 2015), making the long-term trend analysis impossible for this study. Therefore, the results of this study may not necessarily be an overall picture of Japanese publicly competitive research funding.

Sixth, only public competitive funding systems were covered in this study. Public in-house research funding (i.e. budgets related to R&D carried out by national research institutions using operating grants, etc. granted by the national government: about 670 million USD per year[59]) was not considered. Also, private research funding was not to be considered because information regarding these funding is scattered and these data cannot be collected comprehensively. This implies a huge data gap. For example, according to a previous study, the private sector was estimated to contribute nearly three times as much as the public sector in terms of global health R&D funding that meets the needs of low- and middle-income countries [60]. Given the nature of the public competitive funding systems considered in this study are focused more on domestic health issues, this figure is not necessarily an appropriate reference, but can be good information for understanding the size of private resources. Ideally, with due consideration of both public and private finding, more strategic prioritization of health R&D is required in light of the balance with the burden of disease.

To overcome these limitations, national monitoring of health R&D must be strengthened, which could ultimately enable adequate financing for priority areas, aid efficiency and targeting of low resources, and improvement of investment decisions through avoidance of duplication and improved coordination between different funding systems including public and private sectors. Increased transparency would enable funders to be accountable for investments in health R&D. It also makes it easier for researchers to identify research projects that are similar to theirs, eliminate overlap with existing research, and improve their projects.

The importance of monitoring of health R&D is not limited to Japan or at domestic levels. The World Bank's recent Money and Microbes report suggested that governments and development partners in low- and middle-income countries first need to monitor and track all available funding that support health R&D activities in the countries in order to facilitate the rapid development of effective and affordable new discoveries at scale [8]. Recent studies by the World Health Organization’s Global Observatory on Health R&D on the sectoral distribution of health products and biomedical research funding at the global level also warned that today’s product development and funding allocations are not necessarily based on public health needs and evidence [9, 10]. In particular, it has been pointed out that they have not been able to respond to the rapid epidemiological transition from infectious diseases to chronic diseases, with highly increasing burden of non-communicable diseases in low- and middle-income countries. The observatory calls for coordinated health R&D efforts by decision makers around the world based on public health needs; it provides data on where and by whom limited resources are being provided for based on specific areas of health research and product development.

Finally, to verify the accuracy of the GBD disease group mapping using natural language processing, a randomly selected 10% (n = 3,220) projects were independently manually reviewed by six authors (ST, RM, HS, AI, HN, and AK) and the results of manual mapping of text information were compared by two authors. As a result, only 43.8% had the same mapping results for manual reviews and natural language processing; we therefore acknowledge that the methodological uncertainties could not be ruled out in this study. Natural language processing can identify and translate more than 25,000 disease names that appear in text information into ICD 10 codes, but manual review cannot. Therefore, the manual review did not pick up ICD 10 codes for the text information, but assigned each research project directly to the GBD disease groups based on the abstract, title, etc. However, due to the limited text information, the assignment process was very difficult for the manual review, and it was challenging to form consensus and make decisions: each database contained only 500–600 words in Japanese per project (equivalent to about 250–300 words in English). As a future suggestion, it is very impractical to assign disease groups to each research project by manual text reviews. Standardization of individual review and judgment criteria may be difficult with a small amount of information. The principal investigator of a research project, who most understands the scope of the research, can define the research area based on ICD 10 codes, and the research database firmly indexes it. Alternatively, funding sources who allocate research funds (ministries in this study) define research areas based on ICD 10 codes in a comparable manner. Meanwhile, the strength of natural language processing is then that it can process information uniformly in accordance with certain predefined rules. Also, in this study, by redistributing research funds by weighting multiple disease groups by frequency of word occurrence, it was possible to deal with probabilistic (uncertain) decisions that can occur in manual reviews to some extent.

In addition, further improvements in natural language processing are possible. In this study, the mapping of text information to GBD disease groups was based on word frequency. This is the most common and efficient technique, but it also fails to account for the context of textual information. The method of extracting latent topics (bag-of-words) that considers not only words but also contexts enables more robust mapping. Implementing state-of-the-art natural language processing technologies, such as deep learning-based methods (e.g. BERT [Bidirectional Encoder Representations from Transformers], recently published by Google AI Language researchers[61]) is our future task.

### Conclusions

While caution is necessary as this study was not able to consider public in-house funding and the methodological uncertainties could not be ruled out, this analysis may provide a snapshot of the limited alignment between publicly competitive disease-specific funding and disease burden in the country, which calls for greater management over the allocation of scarce health research resources. Faced with competing priorities, the burden of disease data will serve as a crucial, but not the only consideration in aligning Japan's research priorities with public health needs. National monitoring of health R&D for both public and private funding must be strengthened, which could help to make Japan's funding priorities more rationale and transparent. In addition, the algorithms for natural language processing used in this study require continued efforts to improve accuracy.

## Supporting information

S1 Fig Balance of health R&D funding (2015–2016) from MEXT and Japan’s and global DALYs in 2016. (PDF)

S2 Fig Balance of health R&D funding (2015–2016) from MHLW and Japan’s and global DALYs in 2016. (PDF)

S3 Fig Balance of health R&D funding (2015–2016) from AMED and Japan’s and global DALYs in 2016. (PDF)

S1 Table Complete GBD-ICD mapping. (PDF)

S2 Table Estimated weights in Eq (1). (PDF)

S3 Table The estimated health R&D funding (2015–2016) from MEXT by the 22 GBD disease categories. (PDF)

S4 Table The estimated health R&D funding (2015–2016) from MHLW by the 22 GBD disease categories. (PDF)

S5 Table The estimated health R&D funding (2015–2016) from AMED by the 22 GBD disease categories. (PDF)

S6 Table The proportion of the estimated total health R&D funding (2015–2016) by the 22 GBD disease categories, compared to those of Japan’s and global DALYs in 2016. (PDF)

S7 Table The proportion of the estimated health R&D funding (2015–2016) from MEXT by the 22 GBD disease categories, compared to those of Japan’s and global DALYs in 2016. (PDF)

S8 Table The proportion of the estimated health R&D funding (2015–2016) from MHLW by the 22 GBD disease categories, compared to those of Japan’s and global DALYs in 2016. (PDF)

S9 Table The proportion of the estimated health R&D funding (2015–2016) from AMED by the 22 GBD disease categories, compared to those of Japan’s and global DALYs in 2016. (PDF)
