# Supplementary material for: Limited alignment of publicly competitive disease funding with disease burden in Japan
Source: PLoS One. 2020 Feb 10;15(2):e0228542. doi: 10.1371/journal.pone.0228542 (PMC7010241; doi:10.1371/journal.pone.0228542)
Supplement: S1 Fig — (PDF) [file pone.0228542.s001.pdf]

S1 Fig. Balance of health R&D funding (2015–2016) from MEXT and Japan's and global DALYs in 2016.

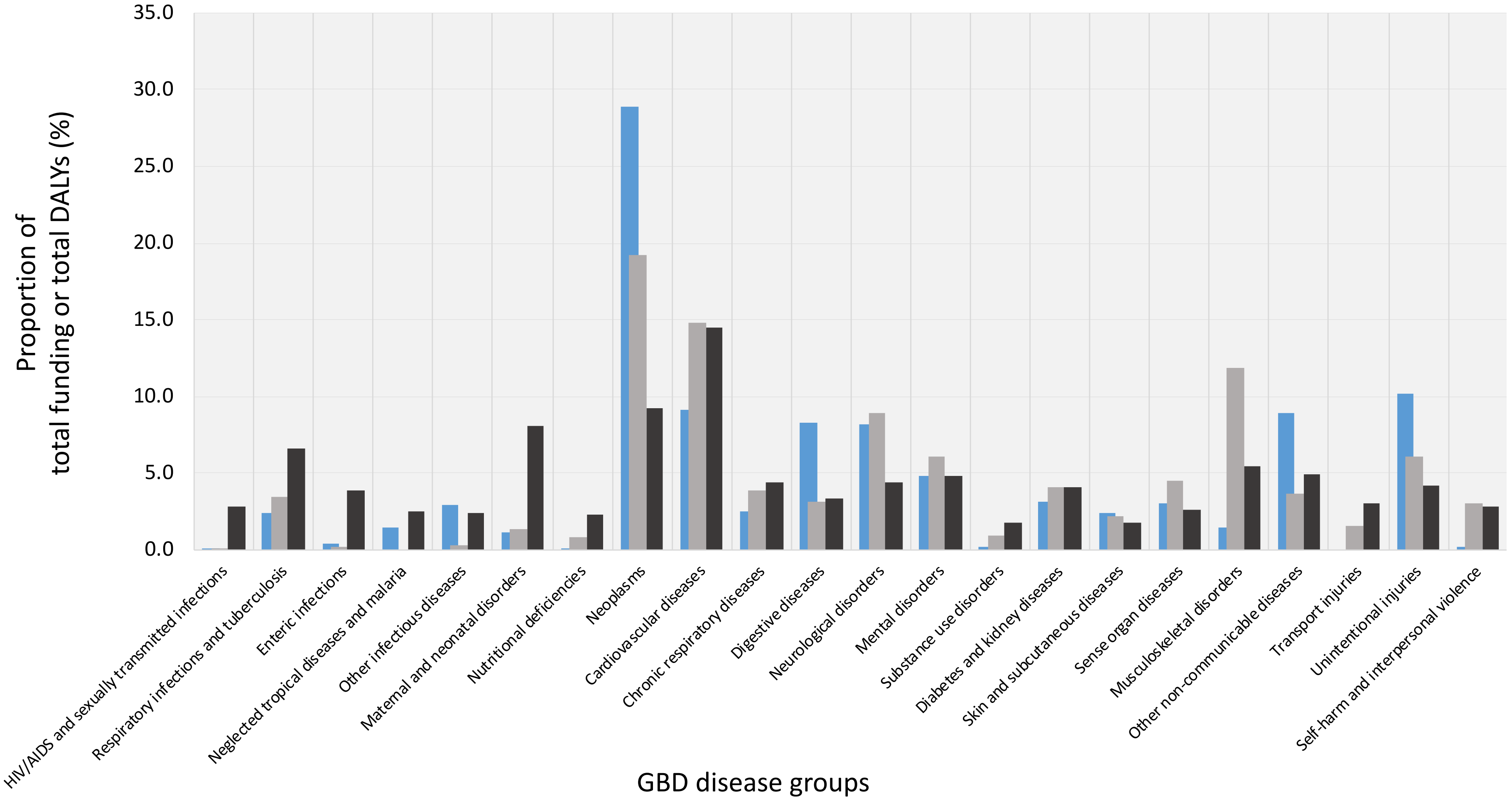

Gray: Japan's DALYs; Black: Global DALYs; Blue: disease-specific R&D funding for 2015 and 2016. MEXT: Ministry of Education, Culture, Sports, Science and Technology. Proportion does not include 'unclassifiable'. Unintentional injuries do not include transport injuries.
