# Supplementary material for: Limited alignment of publicly competitive disease funding with disease burden in Japan
Source: PLoS One. 2020 Feb 10;15(2):e0228542. doi: 10.1371/journal.pone.0228542 (PMC7010241; doi:10.1371/journal.pone.0228542)
Supplement: S1 Table — (PDF) [file pone.0228542.s004.pdf]

S1 Table: Complete GBD-ICD mapping.

GBD code		ICD10
1.	HIV/AIDS and sexually transmitted infections	A50.0–A58, A60.0–A60.9, A63.0–A63.8, B20.0–B24.9, I98.0, K67.0–K67.2, M03.1, M73.0–M73.1
2.	Respiratory infections and tuberculosis	A15.0–A19.9, A48.1, A70, B90.0–B90.9, B97.4–B97.6, H70.0–H70.9, J00–J02.9, J03.0–J03.9, J04.0–J04.2, J05.0–J05.1, J06.0–J06.9, J09–J15.9, J16.0–J16.8, J17.0–J173, J17.8, J18.0–J18.2, J18.8–J18.9, J20.0–J22, J36, K67.3, K93.0, M49.0, M73.8, M90.0, N74.1, P23.0–P23.9, P37.0
3.	Enteric infections	A00.0–A00.9, A01.0–A09.9, A80.0–A80.9, M49.2
4.	Neglected tropical diseases and malaria	A68.0–A68.9, A69.2–A69.9, A75.0–A79.9, A82.0–A82.9, A90–A98.8, B33.0–B33.1, B50.0–B53.8, B54–B57.5, B60.0–B60.8, B65.0–B83.9, K93.1, P37.1, P37.3–37.4
5.	Other infectious diseases	A20.0–A49.9, A48.4, A59.0–A59.9, A64–A65, A69.0–A69.1, A71.0–A74.9, A81.0–A81.9, A83.0–B17.9, B09.0, B19.0–B19.9, B25.0–B33.0, B33.3–B49, B58.0–B59, B64, B85.0–B85.4, B87.0–B89, B91–B92, B94.0–B97.3, B97.7–B97.8, B99, F07.1, G00.0–G00.8, G02.0–G03.8, G04–G05.8, G14, G21.3, G22, G53.0–G53.1, G63.0, G94.0, H06.1, H06.3, H62.0–H62.3, H94.0–H94.8, I00, I02.0–I02.9, I98.1, J99.8, K67.8, K75.3, K76.3, K77.0, M49.1, M49.3, M89.6, M90.1–M90.2, N51.2, P35–P35.9, P37.0, P37.2, P37.5–P37.9
6.	Maternal and neonatal disorders	N96, N98.0–N98.9, O00.0–O94, O96.0–O98.6, O98.8–P04.2, P04.5–P22.9, P24.0–P29.9, P36.0–P36.9, P38.0–P70.1, P70.3–P81.9, P83.0–P83.9, P90–P94.9, P96.0, P96.3–P96.4, P96.8–P96.9
7.	Nutritional deficiencies	D50.1–D50.8, D51–D52.0, D52.8–D53.9, E00.0–E02, E40–E46, E50.0–E64.9, G63.4, H28.1, M12.1
8.	Neoplasms	C00.0–C79.8, C58, C80.0–D48.9, G53.3, G63.1, G94.1, K62.0–K62.1, K63.5, M90.6–M90.7, N60.0–N60.9, N84.0–N84.1, N87.0–N87.9
9.	Cardiovascular diseases	B33.2, G45.0–G46.8, I00–I11.9, I15.0–I13.1, I31.8–I51.9, I38–I41.8, I43.0–I43.8, I60–I67.6, I67.8–I68.2, I69.0–I83.9, I86–I89.0, I89.9–I99, K75.1
10.	Chronic respiratory diseases	D86.0–D86.2, D86.9, G47.3, J30.0–J35.9, J37.0–J70.9, J80–J94.9, J96.0–J98.9
11.	Digestive diseases	B18.0–B18.9, I85.0–I85.9, I98.2, K00.0–K02.4, K02.8–K12.2, K13.0–K42.9, K44.0–K46.9, K55.0–K63.4, K63.8–K76.2, K76.4–K90.9, K920–K92.9, K93.8, M09.1
12.	Neurological disorders	F00.0–F03.9, G00.0, G00.9, G01, G06.0–G13.8, G20, G21.0–G21.2, G21.8–G21.9, G23.0–G44.8, G47.0–G47.2, G47.4, G47.8–G52.9, G54.0–G58.9, G59.8–G72.0, G72.2–G73.7, G90.0–G93.6, G93.8–G93.9, G95.0–G96.9, M33.0–M33.9, M49.4
13.	Mental disorders	F04–F07.0, F07.2–F09, F20.0–F99
14.	Substance use disorders	F10.0–F19.9, G31.2, G72.1, P04.3–P04.4, P96.1, Q86.0, X45, X65
15.	Diabetes and kidney diseases	E10.0–E12.7, E12.9–E13.7, E13.9–E14.7, E14.9–E16.0, G59.0, G632, H28.0, I12.0–I13.9, M90.8, N00.0–N08.8, N13.0–N13.9, N15.0, N17.0–N19, N28.8–N28.9, N32.1–N32.2, N32.8–N33.8, N35.0–N35.9, N39.3–N40, N42.0–N42.2,

		N42.8–N43.4, N46–N48.9, N500–N50.9, N61–N71.9, N73.0–N74.0, N74.2–N74.8, N82.0–N82.9, N84.2–N84.3, N84.8–N86, N840, N88.0–N95.9, N97.0–N97.9, P70.2, Q61.0–Q62.8
16.	Skin and subcutaneous diseases	A46, A66.0–A67.9, B86, D86.3, I89.1–I89.8, L00–L54.8, L56.0–L56.2, L56.4, L57.0–L57.9, L59.0, L59.8–L60.9, L63.0–92.9, L940–L99.8, M72.5–M72.6
17.	Sense organ diseases	H00.0–H22.8, H25.0–H44.9, H47.0–H61.9, H65.0–H93.9, H95.0–H95.9
18.	Musculoskeletal disorders	G636, I27.1, I67.7, J99.0–J99.1, L93.0–L93.2, M00.0–M03.0, M03.2–M09.0, M09.2–M10.0, M10.9–M12.0, M12.2–M32.9, M34.0–M48.9, M50.0–M72.4, M72.8–M73.0, M75.0–M79.6, M79.8–M87.0, M87.2–M99.9
19.	Other non-communicable diseases	D25.0–D26.0, D28.2, D50.0, D50.9, D52.1, D55.0–D84.9, D86.8, D89.0–D89.9, E03.0–E07.9, E15.0–E35.8, E65–E68, E70.0–E87.6, E87.8, E88.0–E89.9, G24.0, G25.1, G25.4, G25.6, G53.2, G63.3, G63.5, G72.0, G93.7, G97.0–G97.9, I68.0, I95.2–I95.3, I97.0–I97.9, J70.0–J70.4, J95.0–J95.9, K43.0–K43.9, K62.7, K91–K91.9, M87.1, N10–N12, N14.0–N16.8, N20.0–N28.1, N29.0–N32.0, N32.3–N32.4, N34.0–N34.3, N36.0–N36.9, N39.0–N39.2, N41.0–N41.9, N44–N45.9, N49.0–N49.9, N72, N75.0–N77.8, N80.0–N81.9, N83.0–N83.9, N99.0–N99.9, P96.0, P96.2, P96.5, Q00.0–Q60.6, Q63.0–Q99.9
20.	Transport injuries	V01.0–V99, Y85.0, Y85.9
21.	Unintentional injuries	L55.0–L55.9, L56.3, L56.8–L56.9, L58.0–L58.9, S00.0–S15.9, S17.0–S17.9, S19.7–T98.3, W00–X44, X46–X59.0, Y40.0–Y84.9, Y86, Y88.0–Y88.3
22.	Self-harm and interpersonal violence	X60–X64, X66–Y09, Y35.0–Y36.9, Y87.0–Y87.1, Y89.0–Y89.1
