# Supplementary material for: Limited alignment of publicly competitive disease funding with disease burden in Japan
Source: PLoS One. 2020 Feb 10;15(2):e0228542. doi: 10.1371/journal.pone.0228542 (PMC7010241; doi:10.1371/journal.pone.0228542)
Supplement: S2 Table — (PDF) [file pone.0228542.s005.pdf]

S2 Table: Estimated weights in Equation (1).

Funding systems	Estimated weights	
	α_1	α_2
MEXT	0.563	0.437
MHLW	0.457	0.543
AMED	0.563	0.437
Total	0.559	0.442

MEXT: Ministry of Education, Culture, Sports, Science and Technology; MHLW: Ministry of Health and Labour Welfare; AMED: Japan Agency for Medical Research and Development
