# Supplementary material for: Limited alignment of publicly competitive disease funding with disease burden in Japan
Source: PLoS One. 2020 Feb 10;15(2):e0228542. doi: 10.1371/journal.pone.0228542 (PMC7010241; doi:10.1371/journal.pone.0228542)
Supplement: S3 Table — (PDF) [file pone.0228542.s006.pdf]

S3 Table: The estimated health R&D funding (2015–2016) from MEXT by the 22 GBD disease categories.

GBD disease groups at level 1	GBD disease groups at level 2		Funding in JPY (million)	Funding in USD* (million)	% of total funding
Communicable, maternal and neonatal conditions and nutritional deficiencies	1.	HIV/AIDS and sexually transmitted infections	52.9	0.5	0.1
	2.	Respiratory infections and tuberculosis	1,111.1	9.7	1.3
	3.	Enteric infections	192.4	1.7	0.2
	4.	Neglected tropical diseases and malaria	663.0	5.8	0.8
	5.	Other infectious diseases	1,344.2	11.7	1.6
	6.	Maternal and neonatal disorders	532.6	4.6	0.6
	7.	Nutritional deficiencies	37.8	0.3	0.0
Non-communicable diseases	8.	Neoplasms	13,226.7	115.1	15.5
	9.	Cardiovascular diseases	4,199.1	36.5	4.9
	10.	Chronic respiratory diseases	1,150.9	10.0	1.4
	11.	Digestive diseases	3,775.2	32.9	4.4
	12.	Neurological disorders	3,725.0	32.4	4.4
	13.	Mental disorders	2,217.2	19.3	2.6
	14.	Substance use disorders	78.1	0.7	0.2
	15.	Diabetes and kidney diseases	1,461.0	12.7	1.7
	16.	Skin and subcutaneous diseases	1,097.8	9.6	1.3
	17.	Sense organ diseases	1,386.7	12.1	1.6
	18.	Musculoskeletal disorders	662.4	5.8	0.8
	19.	Other non-communicable diseases	4,074.0	35.5	4.8
Injuries	20.	Transport injuries	0.0	0.0	0.0
	21.	Unintentional injuries**	4,676.6	40.7	5.5
	22.	Self-harm and interpersonal violence	95.2	0.8	0.1
	23.	Unclassifiable	39,595.2	344.5	46.4

* 114.92 JPY=1 USD; ** Unintentional injuries do not include transport injuries. MEXT: Ministry of Education, Culture, Sports, Science and Technology. Other infectious diseases include meningitis, encephalitis, diphtheria, whooping cough, tetanus, measles, varicella and herpes zoster, acute hepatitis, and other unspecified infectious diseases; other non-infectious diseases include congenital birth defects, urinary diseases and male infertility, gynecological diseases, hemoglobinopathies and hemolytic anemias, endocrine, metabolic, blood, and immune disorders, oral disorders, and sudden infant death syndrome.
