# Supplementary material for: Limited alignment of publicly competitive disease funding with disease burden in Japan
Source: PLoS One. 2020 Feb 10;15(2):e0228542. doi: 10.1371/journal.pone.0228542 (PMC7010241; doi:10.1371/journal.pone.0228542)
Supplement: S4 Table — (PDF) [file pone.0228542.s007.pdf]

S4 Table: The estimated health R&D funding (2015–2016) from MHLW by the 22 GBD disease categories.

GBD disease groups at level 1	GBD disease groups at level 2	Funding in JPY (million)	Funding in USD* (million)	% of total funding
Communicable, maternal and neonatal conditions and nutritional deficiencies	1. HIV/AIDS and sexually transmitted infections	224.0	1.9	2.0
	2. Respiratory infections and tuberculosis	218.3	1.9	1.9
	3. Enteric infections	22.0	0.2	0.2
	4. Neglected tropical diseases and malaria	26.6	0.2	0.2
	5. Other infectious diseases	370.6	3.2	3.3
	6. Maternal and neonatal disorders	27.7	0.2	0.3
	7. Nutritional deficiencies	15.5	0.1	0.1
Non-communicable diseases	8. Neoplasms	501.0	4.4	4.5
	9. Cardiovascular diseases	395.7	3.4	3.5
	10. Chronic respiratory diseases	95.8	0.8	0.9
	11. Digestive diseases	699.0	6.1	6.2
	12. Neurological disorders	812.2	7.1	7.2
	13. Mental disorders	332.0	2.9	3.0
	14. Substance use disorders	79.5	0.7	0.7
	15. Diabetes and kidney diseases	134.4	1.2	1.2
	16. Skin and subcutaneous diseases	90.4	0.8	0.8
	17. Sense organ diseases	379.1	3.3	3.4
	18. Musculoskeletal disorders	110.2	1.0	1.0
	19. Other non-communicable diseases	812.3	7.1	7.2
Injuries	20. Transport injuries	0.0	0.0	0.0
	21. Unintentional injuries**	678.8	5.9	6.0
	22. Self-harm and interpersonal violence	61.9	0.5	0.6
	23. Unclassifiable	5,168.2	45.0	45.9

* 114.92 JPY=1 USD; ** Unintentional injuries do not include transport injuries. MHLW: Ministry of Health and Labour Welfare. Other infectious diseases include meningitis, encephalitis, diphtheria, whooping cough, tetanus, measles, varicella and herpes zoster, acute hepatitis, and other unspecified infectious diseases; other non-infectious diseases include congenital birth defects, urinary diseases and male infertility, gynecological diseases, hemoglobinopathies and hemolytic anemias, endocrine, metabolic, blood, and immune disorders, oral disorders, and sudden infant death syndrome.

oral disorders, and sudden infant death syndrome.
