# Supplementary material for: Limited alignment of publicly competitive disease funding with disease burden in Japan
Source: PLoS One. 2020 Feb 10;15(2):e0228542. doi: 10.1371/journal.pone.0228542 (PMC7010241; doi:10.1371/journal.pone.0228542)
Supplement: S5 Table — (PDF) [file pone.0228542.s008.pdf]

S5 Table: The estimated health R&D funding (2015–2016) from AMED by the 22 GBD disease categories.

GBD disease groups at level 1	GBD disease groups at level 2		Funding in JPY (million)	Funding in USD* (million)	% of total funding
Communicable, maternal and neonatal conditions and nutritional deficiencies	1.	HIV/AIDS and sexually transmitted infections	65.6	0.6	0.0
	2.	Respiratory infections and tuberculosis	5,395.1	46.9	2.2
	3.	Enteric infections	838.1	7.3	0.3
	4.	Neglected tropical diseases and malaria	2,241.5	19.5	0.9
	5.	Other infectious diseases	4,151.8	36.1	1.7
	6.	Maternal and neonatal disorders	315.0	2.7	0.1
	7.	Nutritional deficiencies	12.2	0.1	0.0
Non-communicable diseases	8.	Neoplasms	34,870.0	303.4	14.1
	9.	Cardiovascular diseases	8,940.8	77.8	3.6
	10.	Chronic respiratory diseases	1,246.1	10.8	0.5
	11.	Digestive diseases	8,636.7	75.2	3.5
	12.	Neurological disorders	16,094.3	140.0	6.5
	13.	Mental disorders	6,087.9	53.0	2.5
	14.	Substance use disorders	122.6	1.1	0.1
	15.	Diabetes and kidney diseases	1,893.0	16.5	0.8
	16.	Skin and subcutaneous diseases	2,131.6	18.5	0.9
	17.	Sense organ diseases	3,058.2	26.6	1.2
	18.	Musculoskeletal disorders	853.6	7.4	0.3
	19.	Other non-communicable diseases	12,257.8	106.7	5.0
Injuries	20.	Transport injuries	0.0	0.0	0.0
	21.	Unintentional injuries**	9,120.6	79.4	3.7
	22.	Self-harm and interpersonal violence	34.6	0.3	0.0
	23.	Unclassifiable	129,137.8	1,123.7	52.2

* 114.92 JPY=1 USD; ** Unintentional injuries do not include transport injuries. AMED: Japan Agency for Medical Research and Development. Other infectious diseases include meningitis, encephalitis, diphtheria, whooping cough, tetanus, measles, varicella and herpes zoster, acute hepatitis, and other unspecified infectious diseases; other non-infectious diseases include congenital birth defects, urinary diseases and male infertility, gynecological diseases, hemoglobinopathies and hemolytic anemias, endocrine, metabolic, blood, and immune disorders, oral disorders, and sudden infant death syndrome.
