# Supplementary material for: Limited alignment of publicly competitive disease funding with disease burden in Japan
Source: PLoS One. 2020 Feb 10;15(2):e0228542. doi: 10.1371/journal.pone.0228542 (PMC7010241; doi:10.1371/journal.pone.0228542)
Supplement: S8 Table — (PDF) [file pone.0228542.s011.pdf]

S8 Table: The proportion of the estimated health R&D funding (2015–2016) from MHLW by the 22 GBD disease categories, compared to those of Japan's and global DALYs in 2016

GBD disease groups at level 1	GBD disease groups at level 2	% of total funding	Japan		Global	
			% of total DALYs	p-value	% of total DALYs	p-value
Communicable, maternal and neonatal conditions and nutritional deficiencies	1. HIV/AIDS and sexually transmitted infections	3.7	0.1	<0.01	2.8	1.00
	2. Respiratory infections and tuberculosis	3.6	3.5	1.00	6.6	<0.05
	3. Enteric infections	0.4	0.2	1.00	3.8	<0.001
	4. Neglected tropical diseases and malaria	0.4	0.0	0.85	2.5	<0.01
	5. Other infectious diseases	6.1	0.3	<0.01	2.4	<0.01
	6. Maternal and neonatal disorders	0.5	1.4	0.44	8.1	<0.001
	7. Nutritional deficiencies	0.3	0.8	1.00	2.4	<0.01
Non-communicable diseases	8. Neoplasms	8.2	19.2	<0.01	9.2	1.00
	9. Cardiovascular diseases	6.5	14.8	<0.01	14.5	<0.001
	10. Chronic respiratory diseases	1.6	3.9	<0.05	4.4	<0.01
	11. Digestive diseases	11.5	3.1	<0.01	3.4	<0.001
	12. Neurological disorders	13.3	8.9	<0.05	4.4	<0.001
	13. Mental disorders	5.5	6.1	1.00	4.8	1.00
	14. Substance use disorders	1.3	0.9	1.00	1.7	1.00
	15. Diabetes and kidney diseases	2.2	4.1	0.22	4.1	0.13
	16. Skin and subcutaneous diseases	1.5	2.2	1.00	1.7	1.00
	17. Sense organ diseases	6.2	4.5	0.85	2.6	<0.01
	18. Musculoskeletal disorders	1.8	11.9	<0.01	5.4	<0.001
	19. Other non-communicable diseases	13.3	3.7	<0.01	4.9	<0.001
Injuries	20. Transport injuries	0.0	1.6	<0.01	3.0	<0.001
	21. Unintentional injuries*	11.2	6.1	<0.01	4.3	<0.001
	22. Self-harm and interpersonal violence	1.0	3.0	<0.05	2.9	<0.05

* Unintentional injuries do not include transport injuries. MHLW: Ministry of Health and Labour Welfare. Other infectious diseases include meningitis, encephalitis, diphtheria, whooping cough, tetanus, measles, varicella and herpes zoster, acute hepatitis, and other unspecified infectious diseases; other non-infectious diseases include congenital birth defects, urinary diseases and male infertility, gynecological diseases, hemoglobinopathies and hemolytic anemias, endocrine, metabolic, blood, and immune disorders, oral disorders, and sudden infant death syndrome.
