# Supplementary material for: Limited alignment of publicly competitive disease funding with disease burden in Japan
Source: PLoS One. 2020 Feb 10;15(2):e0228542. doi: 10.1371/journal.pone.0228542 (PMC7010241; doi:10.1371/journal.pone.0228542)
Supplement: S9 Table — (PDF) [file pone.0228542.s012.pdf]

S9 Table: The proportion of the estimated health R&D funding (2015–2016) from AMED by the 22 GBD disease categories, compared to those of Japan's and global DALYs in 2016.

GBD disease groups at level 1	GBD disease groups at level 2	% of total funding	Japan		Global	
			% of total DALYs	p-value	% of total DALYs	p-value
Communicable, maternal and neonatal conditions and nutritional deficiencies	1. HIV/AIDS and sexually transmitted infections	0.1	0.1	1.00	2.8	<0.001
	2. Respiratory infections and tuberculosis	4.6	3.5	0.86	6.6	0.49
	3. Enteric infections	0.7	0.2	0.42	3.8	<0.001
	4. Neglected tropical diseases and malaria	1.9	0.0	<0.001	2.5	1.00
	5. Other infectious diseases	3.5	0.3	<0.001	2.4	0.49
	6. Maternal and neonatal disorders	0.3	1.4	0.13	8.1	<0.001
	7. Nutritional deficiencies	0.0	0.8	0.13	2.4	<0.001
Non-communicable diseases	8. Neoplasms	29.5	19.2	<0.001	9.2	<0.001
	9. Cardiovascular diseases	7.6	14.8	<0.001	14.5	<0.001
	10. Chronic respiratory diseases	1.1	3.9	<0.05	4.4	<0.01
	11. Digestive diseases	7.3	3.1	<0.01	3.4	<0.01
	12. Neurological disorders	13.6	8.9	<0.01	4.4	<0.001
	13. Mental disorders	5.1	6.1	0.14	4.8	1.00
	14. Substance use disorders	0.1	0.9	0.19	1.7	<0.01
	15. Diabetes and kidney diseases	1.6	4.1	<0.05	4.1	<0.05
	16. Skin and subcutaneous diseases	1.8	2.2	1.00	1.7	1.00
	17. Sense organ diseases	2.6	4.5	0.42	2.6	1.00
	18. Musculoskeletal disorders	0.7	11.9	<0.001	5.4	<0.001
	19. Other non-communicable diseases	10.4	3.7	<0.001	4.9	<0.001
Injuries	20. Transport injuries	0.0	1.6	<0.01	3.0	<0.001
	21. Unintentional injuries*	7.7	6.1	1.00	4.3	0.08
	22. Self-harm and interpersonal violence	0.0	3.0	<0.001	2.9	<0.001

* Unintentional injuries do not include transport injuries. AMED: Japan Agency for Medical Research and Development. Other infectious diseases include meningitis, encephalitis, diphtheria, whooping cough, tetanus, measles, varicella and herpes zoster, acute hepatitis, and other unspecified infectious diseases; other non-infectious diseases include congenital birth defects, urinary diseases and male infertility, gynecological diseases, hemoglobinopathies and hemolytic anemias, endocrine, metabolic, blood, and immune disorders, oral disorders, and sudden infant death syndrome.
